# Economic Evaluation of a Tai Ji Quan Intervention to Reduce Falls in People With Parkinson Disease, Oregon, 2008–2011

**DOI:** 10.5888/pcd12.140413

**Published:** 2015-07-30

**Authors:** Fuzhong Li, Peter Harmer

**Affiliations:** Author Affiliation: Peter Harmer, Willamette University, Salem, Oregon.

## Abstract

**Introduction:**

Exercise is effective in reducing falls in people with Parkinson disease. However, information on the cost effectiveness of this approach is lacking. We conducted a cost-effectiveness analysis of Tai Ji Quan for reducing falls among patients with mild-to-moderate Parkinson disease.

**Methods:**

We used data from a previous intervention trial to analyze resource use costs related to intervention delivery and number of falls observed during a 9-month study period. Cost effectiveness was estimated via incremental cost-effectiveness ratio (ICER) in which Tai Ji Quan was compared with 2 alternative interventions (Resistance training and Stretching) on the primary outcome of per fall prevented and the secondary outcome of per participant quality-adjusted life years (QALY) gained. We also conducted subgroup and sensitivity analyses.

**Results:**

Tai Ji Quan was more effective than either Resistance training or Stretching; it had the lowest cost and was the most effective in improving primary and secondary outcomes. Compared with Stretching, Tai Ji Quan cost an average of $175 less for each additional fall prevented and produced a substantial improvement in QALY gained at a lower cost. Results from subgroup and sensitivity analyses showed no variation in cost-effectiveness estimates. However, sensitivity analyses demonstrated a much lower ICER ($27) when only intervention costs were considered.

**Conclusion:**

Tai Ji Quan represents a cost-effective strategy for optimizing spending to prevent falls and maximize health gains in people with Parkinson disease. While these results are promising, they warrant further validation.

## Introduction

Exercise offers physical benefits for people with Parkinson disease (PD), including improved balance and lower-extremity strength and reductions in the rate of falls (1–4). A recent trial found that Tai Ji Quan training, which has reduced falls and risk of falling in older adults (5,6), also helped reduce the incidence of falls in people with PD (7).

However, there has been limited information on the cost effectiveness of exercise-based programs for preventing falls (3). Results of a previous study suggest that, although there was no difference between exercise and control groups in the incidence of falls (8), exercise may be a cost-effective strategy for reducing falls for people with PD (9). Given that the economic costs of injurious falls in people with PD add to the already increased financial burden on families and health care systems (10,11), additional economic studies are needed to determine the cost effectiveness of exercise-based interventions to make optimal use of community and health care resources in fall prevention efforts.

We present a secondary data analysis on the economic characteristics of a Tai Ji Quan program implemented in a previously completed exercise trial (7). Specifically, we conducted a cost-effectiveness analysis of the Tai Ji Quan program compared with low-impact Stretching and Resistance training programs on the primary outcome of falls prevented and the secondary outcome of quality-adjusted life years (QALY) gained.

## Methods

The original study design involved a randomized controlled trial conducted from 2008 through 2011 (7). The trial consisted of a 6-month active intervention period (with 60-minute classes conducted 2 times weekly) and a 3-month postintervention follow-up and compared Tai Ji Quan and Resistance training to a Stretching control. The study protocol was approved by the institutional review board of the Oregon Research Institute, and written informed consent was obtained from all participants.

The study involved individuals who were diagnosed with mild to moderate PD, defined as stages 1 through 4 on the Hoehn-Yahr scale (12). Participants were recruited through newspaper advertisements, promotions through local support groups and medical clinics, and referrals from health care providers and were drawn from 4 different cities in Oregon. Eligibility required that participants had 1) a clinical diagnosis of PD and were aged from 40 to 85 years; 2) at least 1 or 2 motor symptoms of tremor, rigidity, postural stability, or bradykinesia for at least 1 limb; 3) stable medication; 4) the ability to stand unaided and walk; 5) medical clearance; and 6) willingness to accept any of the 3 interventions. Individuals were excluded if they were currently participating in any active interventions, had signs of cognitive impairment (13), or had physical conditions that would prevent them from engaging in exercise safely.

### Interventions

Almost all intervention classes (96%) were conducted in community settings (eg, community centers, churches). Class size ranged from 6 to 15 people, with an average 1:8 instructor-to-participant ratio. No specific transportation arrangements or remuneration were provided that enabled participants to attend the intervention classes; 97% provided their own transportation. Both Resistance training and Stretching instructors were certified exercise/fitness instructors through professional organizations (eg, American College of Sports Medicine; American Council on Exercise). Tai Ji Quan instructors were trained and certified by the principal author of the study (F.L.).

Participants in the Tai Ji Quan group began practicing a set of 6 adapted Tai Ji Quan movements that were progressively integrated into a complete 8-form routine (14,15). Exercises were primarily targeted at balance and lower-extremity strength, focusing on training symmetrical and diagonal movements such as ankle sway, controlled displacement of the center of mass over the base of support (16), rotational weight shifting at the torso, and anterior–posterior and lateral stepping.

Participants in the Resistance training group received a progressive strength-training protocol that involved body weight and additional external weights. Training focused primarily on lower-extremity strength exercises such as forward/side stepping, squats, forward/side lunges, and heel and toe raises. Additional moderate-intensity training stimulus using weighted vests and ankle weights was added at week 10; weighted vest resistance was initially set at 1% of body weight and progressively increased until 5% of body weight was achieved. Ankle weights started at 0.45 kg per limb and were gradually increased to 1.36 kg. The exercises were performed in 1 to 3 sets of 10 to 15 repetitions.

Participants in the Stretching group were provided with an exercise regimen that encompassed various seated and standing stretches involving the upper body (neck, upper back, shoulder, chest, and arms) and lower extremities (quadriceps, hamstring/calf, and hip), using gentle joint extension and flexion and trunk rotation. Breathing that emphasized inhaling and exhaling to maximum capacity and relaxation of major muscles was also included.

### Outcome measures

The primary outcome was the number of falls prevented over the 9-month study period, beginning at baseline. Falls were defined as when a person lands on the floor or the ground or falls and hits objects like stairs or pieces of furniture, by accident (5). A falls calendar was given to each participant at baseline to record his or her daily falls information and was collected by research staff monthly throughout the 9-month period or until the participant withdrew from the study. Research staff members called participants monthly to verify reported falls information.

The secondary outcome was health-related quality of life, as defined by selected items from the Parkinson’s Disease Questionnaire (PDQ-8) (17), collected at baseline and again at 9-month follow-up. PDQ-8 items used were dimensions of mobility (Item 1), activities of daily living (Item 2), depression (Item 3), and bodily discomfort (Item 7); each item was scored on a 0 to 4 scale (never, occasionally, sometimes, often, always).

Each of the 4 PDQ-8 item scores was converted into a range of points, from 1 (no problems) to 3 (severe problems/inability), that was comparable to the EQ-5D measure of QALY (18). Each of the resulting points for each item was then weighted by a constant value (19), and all items were combined into a weighted single health utility (with 1 representing the “best possible health” state and 0 representing the “worst possible health” state) to estimate the QALY. We calculated the QALY by using the constructed health utility values at baseline and 9-month follow-up.

### Resource use costs and valuation

All costs were documented by the project staff by using monthly fiscal reports and expense-tracking sheets. We took a societal perspective to include resource use costs required to implement an exercise program for people with PD in community practice. Our valuation covered costs incurred during the 6-month intervention and the 3-month follow-up (a total of 9 months) and were divided into 2 areas: intervention- and nonintervention-related costs.

Intervention-related costs were mainly considered variable because they depended on the number of participants (recruited) and classes (conducted). They were categorized as 1) program promotional costs, 2) participant recruitment costs plus overhead, and 3) class delivery-related costs. Costs associated with developing the exercise programs were excluded since they were expenses that occurred before the trial. Also excluded were costs associated with the trial, including administration, data collection, and outcome assessments. The cost of promotion included printed class flyers placed in community and senior centers and newspaper advertisements. Participant recruitment costs included the time and effort research staff spent screening potential participants to see if they met eligibility criteria and enrolling eligible participants in the study. Costs related to delivery of the intervention class included fees for the class instructors, instructor liability insurance coverage, classroom rental, exercise equipment (eg, weights), and class-related printed materials.

Nonintervention-related cost included participant travel time, costs of medication use, physical therapy, and medical treatment of falls. These item costs were calculated as follows. For travel expenses to classes, the Internal Revenue Service 2011 mileage rate was applied to each participant’s home-to-class location distance, taking into account his or her weekly class attendance. The self-reported number of major PD medications (ie, carbidopa/levodopa, Requip/Mirapex) taken during the 9-month period was counted for each participant and converted into a price value (number of medications × average price × 9 months), using information derived from GoodRx in 2015 (www.goodrx.com/parkinson-s-disease/drugs). The number of physical therapy sessions reported by participants was multiplied by $79.86, a value based on salary estimates obtained from the US Occupational Outlook Handbook (www.bls.gov/oof/home.htm). Using the average direct medical cost of $11,502 per fall in 2012 (20), fall-related treatment costs were estimated on the basis of participant reports of injurious falls that required a medical treatment (2 falls occurred in the Stretching group during the 3-month postintervention follow-up).

By using a 3% social discount rate, all unit costs (in US dollars) were calculated for the financial year 2011 (base year).

### Data analysis

Descriptive statistics and between-group differences on falls and QALY scores were calculated. No falls were assumed to have occurred for participants who dropped out of the study, and the last-observation-carried-forward method was used for missing data on the PDQ-8.

The economic analyses compared resource use costs and intervention effectiveness between Tai Ji Quan versus Stretching and between Resistance training versus Tai Ji Quan via a cost-effectiveness analysis based on an incremental cost-effectiveness ratio (ICER), defined as the difference in incremental costs (ΔC between Tai Ji Quan and Stretching and between Resistance training and Tai Ji Quan) divided by the difference in intervention effect (ΔE [ie, number of falls prevented] per participant per QALY gained). Within a net-benefit framework, we also calculated the net health benefit on the study outcomes, defined as ΔE − ΔC ÷ λ, where λ indicates a willingness-to-pay value (ie, what society is willing to pay for a new therapy). We chose 2 values for willingness to pay: $14,306 and $21,270 (in 2013 dollars), which represented averaged Medicare costs per fall (21). A positive net health benefit value (>0) indicates the cost of the Tai Ji Quan intervention to be less than the value of the additional benefit achieved; therefore, it should be considered cost effective (22).

The analytic horizon in this study was confined to the time frame of the 9-month study. In choosing a baseline comparator, it was reasoned that the Stretching exercise would provide an estimate of how cost effective Tai Ji Quan was compared with a minimally involved physical activity program of the type commonly implemented in the general community. Similarly, comparing Resistance training, the next best alternative and the one most often studied in the PD exercise literature, with Tai Ji Quan would provide information of the relative cost effectiveness of Tai Ji Quan against a mainstream exercise approach.

Subgroup analyses were conducted on 3 variables that may have affected the outcome: 1) high adherence (≥75%), 2) history of falls at baseline (those who reported 1 or more falls before the beginning of the intervention), and 3) disease severity at baseline (equal or greater than stage 2 on the Hoehn-Yahr scale [12]).

To address uncertainty in the estimates of falls prevented, we conducted 1-way sensitivity analysis by considering plausible variations in costs and effect sizes that may be subject to change if the Tai Ji Quan program were implemented in different community settings or economic situations or both. In the first sensitivity analysis, we considered a partial case where nonintervention-related costs were removed. In the second analysis, we added additional program costs, including expenses for instructor training workshops ($3,000), refresher courses ($4,500 total for up to 3 refresher courses), and an annual licensing fee of $3,000, which would be expected in community-based implementation. In the third analysis, we considered a full case in which cost adjustments for Tai Ji Quan were made either in a worst-case scenario (where program implementation expenses increase by 5% and 10%) or a best-case scenario (where expenses decrease by 5% and 10%). Finally, we calculated ICER by varying the effect sizes of the Tai Ji Quan group on the basis of confidence intervals of the observed number of falls at 95% and 90%.

## Results

The baseline characteristics of the study population are described elsewhere (5). In brief, the 3 intervention groups were well balanced with respect to participant demographic and clinical profiles. A total of 176 participants (90%) completed their assigned interventions. There were no observed differences in the baseline demographic variables or primary outcomes between participants who completed the trial (ie, finished the assigned intervention; n = 176) and those who did not (ie, drop-outs; n = 19). Intervention adherence, defined as the percentage of sessions attended, was approximately 77% overall (of the total 48 sessions); 70% of participants attended at least 75% of the sessions (ie, 36 sessions of 48). The Tai Ji Quan group had an average adherence of 77%; the Resistance training group, 77%; and the Stretching group, 79%.

### Falls and QALY

Information on the history of falls in the 6 months before study entry as reported by study participants indicated no between-group difference at baseline. Over the 9-month study period, 526 falls were documented on the basis of participant monthly self-reports (Table 1). Participants in Tai Ji Quan had the lowest average number of falls (*P* = .01) and incidence rate (per 100 person-months) (*P* = .005) compared with either Stretching or Resistance training.

**Table 1:** Descriptive Statistics on Falls and PDQ-8–Derived Utilities Across the 9-Month Study Period of a Tai Ji Quan Intervention to Reduce Falls, Oregon, 2008–2011

Measure	Intervention Group (N = 195)		
	Tai Ji Quan (n = 65)	Resistance Training (n = 65)	Stretching (n = 65)
**Falls**			
No. of fallers^a^	22	32	37
No. of total falls	87	172	267
Mean no. (SD) of falls	1.33 (2.52)	2.65 (4.65)	4.11 (7.44)
Fall incident rate per 100 person-months^b^	12	23	38
**PDQ-8–derived utilities**			
Baseline utility, mean (SD)	0.63 (0.33)	0.66 (0.26)	0.65 (0.29)
9-Month follow-up utility, mean (SD)	0.74 (0.26)	0.63 (0.22)	0.59 (0.31)

Abbreviation: PDQ-8, The Parkinson’s Disease Questionnaire (17).

a Falls data were calculated on the basis of information from falls calendars provided by the participants.

b Defined as the number of falls that occurred during the study period divided by the total follow-up time in months (person-months of follow-up).

Table 1 also shows baseline and 9-month PDQ-8–derived EQ-5D equivalent scores used to calculate QALY. Tai Ji Quan showed a significant change in the QALY scores from baseline compared with Resistance training or Stretching (*P* < .05). Inspection of pre-to-post change in scores indicates that there was a significant improvement from baseline for Tai Ji Quan (mean difference = 0.11) but not for Resistance training (mean difference = −0.03, *P* = .56) or Stretching (mean difference = −0.06, *P* = .28). No significant difference between Resistance training and Stretching was observed.

### Costs effectiveness and net health benefit

Overall, resource use costs for the study totaled $281,289, or an average of $1,443 per participant, to deliver programs to 195 participants (with an average class size of 8 participants) across the 24-week trial period (Table 2). Of the 3 programs, Tai Ji Quan had the lowest total resources use costs ($80,441) and average cost per person ($1,238) compared with Stretching ($111,896; $1,721) and Resistance ($88,952; $1,368), respectively.

**Table 2:** Categories and Costs of Interventions Applied in the Economic Evaluation of a Tai Ji Quan Intervention to Reduce Falls, Oregon (Base Year: 2011)

Categories of Cost	Intervention Group (N = 195)			Total Cost, $
	Tai Ji Quan (n = 65)	Resistance Training (n = 65)	Stretching (n = 65)	
**Intervention**				
Program promotion	$750	$750	$750	$2,250
Patient recruitment^a^	$22,500	$22,500	$22,500	$67,500
Overhead^b^	$12,555	$12,555	$12,555	$37,665
Class instruction (teaching and training)^c^	$20,160	$20,160	$20,160	$60,480
Instructor liability insurance^d^	$525	$525	$525	$1,575
Classroom rental^e^	$13,440	$13,440	$13,440	$40,320
Intervention training equipment^f^	—	$6,876	$4,875	$11,751
Instructional material printing	$270	$270	$270	$810
**Nonintervention**				
Travel costs incurred by participants^g^	$3,634	$3,584	$3,606	$10,824
Physical therapy^h^	$1,916	$2,635	$4,522	$9,073
Medication costs^i^	$12,150	$13,905	$16,065	$42,120
Injury falls medical treatment^j^	0	0	$23,004	$23,004
**Total** k	$80,441	$88,952	$111,896	$281,289
**Average cost per participant**	$1,238	$1,368	$1,721	$1,443

Abbreviation: —, not applicable.

a Primarily salaries of research assistants.

b Included computers, fax, telephone, and office supplies.

c Paid at a $40/h rate for each instructor.

d Paid at $175 per insurance policy (as fitness trainer).

e Paid at an average rental rate of $20 per class session.

f Costs related to weight vests and ankle weights (Resistance training ) or chairs (Stretching).

g 2011 rate of 19 cents/mile/person (the rate per mile driven for medical purposes); http://www.irs.gov/uac/IRS-Announces-2011-Standard-Mileage-Rates.

h Number of physical therapy sessions reported by participants multiplied by $79.86, using the salary estimates obtained from the US Occupational Outlook Handbook (www.bls.gov/oof/home.htm).

i Number of major antiparkinsonian medications (ie, carbidopa/levodopa, Requip/Mirapex) reported as being used by participants and converted into a price value as number of medications × average price of $15 per month × 9 months.

j Based on the average direct medical cost of $11,502 per fall in 2012 (20).

k Discounted at a 3% annual rate.

Compared with Stretching, the Tai Ji Quan program had lower cost and greater effectiveness in both the incremental number of falls prevented (n = 180) and QALY gained (.13). In contrast, compared with Tai Ji Quan, the Resistance training program was less effective and had greater costs for both reducing falls (there were 85 more falls in the Resistance training group than in the Tai Ji Quan group) and improving QALY (−.11, a decrease in QALY in comparison to Tai Ji Quan). In terms of incremental cost effectiveness, Tai Ji Quan showed an average reduction of $175 per additional fall prevented and $3,394 per participant per additional QALY gained compared with Stretching. Resistance training showed an average of $100 per additional fall prevented and $1,236 per participant per additional QALY gained. Because of the inferiority of the Resistance training program in cost (ie, more costly) and effectiveness (less effective), it is removed from the subsequent analyses.

For Tai Ji Quan relative to Stretching, the derived net health benefit for the 2 willingness-to-pay values were positive and greater than zero for the outcome of falls: 180 − (−$31,455 ÷ $14,306) = 182 and 180 − (−$31,455 ÷ $21,270) = 181. The values also were positive and greater than zero for QALY: 0.13 − ($483 ÷ $14,306) = 0.10 and 0.13 − ($483 ÷ $21,270) = 0.11.

### Subgroup and sensitivity analyses

Results from the subgroup analyses are shown in Table 3. The analyses excluded the QALY outcome because of its negative value on the incremental cost-effectiveness ratio. When considering high adherence, history of falls, and disease severity, Tai Ji Quan continued to show reduced cost and increased effectiveness in reducing falls relative to Stretching.

**Table 3:** Subgroup Analyses of a Tai Ji Quan Intervention to Reduce Falls, Oregon, 2008–2011

Analysis	Subgroup Analyses			
	Cost	No. of Falls	Incremental Cost^a^	Incremental Effect^b^
**High Adherence (≥75% In-Class Attendance)**				
Stretching	$79,955	206	—	—
Tai Ji Quan	$51,209	59	−$28,746	147
**History of falls at baseline**				
Stretching	$57,151	216	—	—
Tai Ji Quan	$32,572	60	−$24,579	156
**Disease severity at baseline (≥2.0 on Hoehn-Yahr scale [** 12 **])**				
Stretching	$80,447	219	—	—
Tai Ji Quan	$69,968	85	−$10,479	134

Abbreviation: —, not applicable.

a Indicating lower resource use costs in comparison to Stretching.

b Indicating the number of falls prevented in comparison to Stretching.

Results from the sensitivity analyses indicated that 1) excluding nonintervention costs reduced the ICER for Tai Ji Quan from $175 to $27 (a difference of $148) relative to Stretching, 2) including additional program training costs resulted in an average ICER cost of $116 per additional fall prevented, and 3) adjusting resource use costs of the Tai Ji Quan program (5% and 10% up and down) had no substantial impact on the cost-effectiveness of falls averted (5% increase was $152 and 10% increase was $130; 5% decrease was $176 and 10% decrease was $219). The ICERs remained robust when effect sizes were varied at the 2 confidence intervals selected (95%, $173–$177 less per additional fall prevented; 90%, $174–$176 less per additional fall prevented).

## Discussion

The main finding from this study is that 2-times-per-week Tai Ji Quan training for 6 months, with an adherence rate of 77%, resulted in a reduced cost of $175 per additional fall prevented relative to a Stretching exercise program. Additional net health benefit analyses show support for the cost effectiveness of Tai Ji Quan. This is in contrast to incremental cost effectiveness observed in the comparison between the next best exercise program — Resistance training — versus Tai Ji Quan, with the former being less effective and more expensive in reducing falls. Results of the QALY measure consistently showed Tai Ji Quan as being highly cost effective compared with both Stretching and Resistance training.

Findings from subgroup analyses also show consistent support for the cost effectiveness of Tai Ji Quan in comparison to Stretching in reducing falls. Additionally, sensitivity analyses with varying case scenarios showed robustness in cost-effectiveness estimates in terms of cost per fall averted by Tai Ji Quan relative to Stretching. However, a reduction in cost effectiveness was evident when nonintervention-related costs were removed; a change in ICER from $175 to $27 indicated that the Tai Ji Quan intervention was influenced by nonintervention-related costs. After excluding nonintervention costs, there was essentially very little difference in intervention costs between the 2 interventions ($70,200 for Tai Ji Quan versus $75,075 for Stretching) and the incremental cost per fall prevented or QALY gained was largely being driven by the significant reduction in incidence of falls and improvement in QALY attributed to Tai Ji Quan.

Estimates indicate that 20% to 30% of falls in community-dwelling older adults result in injury (23); an average health care cost of a fall injury is approximately $11,000 (20). With a total of 180 falls prevented (the difference between Tai Ji Quan and Stretching) and the assumption that 36 to 54 of these falls could have led to emergency department visits that would cumulatively cost between $396,000 and $594,000 for medical care, our ICER of $175 (less per each additional fall prevented) and other ICERs derived under different scenarios make a clear case for the potential for high cost-savings with the Tai Ji Quan program. In this study we also considered willingness to pay for preventing a fall within the net benefit framework using Medicare costs for treating fall-related injuries and demonstrated the cost effectiveness of Tai Ji Quan relative to Stretching. The combined findings provide information for health planners and decision makers regarding funding decisions on fall prevention programs for people with PD.

The study has limitations. First, like most studies, falls information in this study was gathered primarily on the basis of self-reports, which are subject to recall bias and may not have captured data on all falls despite monthly verification telephone calls made by the research staff. Second, long-term effects were not estimated. Given that PD worsens over time, the extent of the cost effectiveness of Tai Ji Quan training in providing long-term health benefits remains to be determined.

This Tai Ji Quan balance training program delivered in community settings was able to reduce the incidence of falls among people with PD at a lower cost compared with both a Stretching and a Resistance training protocol. Study findings are preliminary but provide a basis for future cost-effectiveness studies with improved methodology to substantiate the economic value of this alternative exercise program for preventing falls in people with PD.
